# A comparative study on the physicochemical and biological stability of IgG_1_ and monoclonal antibodies during spray drying process

**DOI:** 10.1186/2008-2231-22-31

**Published:** 2014-03-18

**Authors:** Vahid Ramezani, Alireza Vatanara, Abdolhossein Rouholamini Najafabadi, Mohammad Ali Shokrgozar, Alireza Khabiri, Mohammad Seyedabadi

**Affiliations:** 1 Department of Pharmaceutics, Faculty of Pharmacy, Tehran University of Medical Sciences, Tehran, Iran; 2 Department of Pharmaceutics, Faculty of Pharmacy, Shahid Sadoughi University of Medical Sciences, Yazd, Iran; 3 National Cell Bank of Iran, Pasteur Institute of Iran, Tehran, Iran; 4 Department of Mycology, Pasteur Institute of Iran, Tehran, Iran; 5 Department of Molecular Imaging, The Persian Gulf Biomedical Sciences Research Institute, Bushehr University of Medical Sciences, Bushehr, Iran

**Keywords:** Antibody, Trastuzumab, Spray drying, Trehalose, Hydroxypropyl beta cyclodextrin, Beta cyclodextrin

## Abstract

**Background:**

The main concern in formulation of antibodies is the intrinsic instability of these labile compounds. To evaluate the physicochemical stability of antibody in dry powder formulations, physical stability of IgG_1_ and a monoclonal antibody (trastuzumab) during the spray drying process was studied in a parallel study and the efficacy of some sugar based excipients in protection of antibodies was studied.

**Results:**

The SDS-PAGE analysis showed no fragmentation of antibodies after spray drying in all formulations. The secondary structure of antibodies contained 40.13 to 70.19% of β structure in dry state. Also, CD spectroscopy showed the similar secondary structure for trastuzumab after reconstitution in water. ELISA analysis and cell culture studies were conducted in order to evaluate bioactivity of monoclonal antibody. Formulations containing combination of excipients provided maximum tendency of trastuzumab to attach to the ELISA antigen (86.46% ± 2.3) and maximum bioactivity when incubated with SKBr_3_ cell line (the cell viability was decreased to 65.99% ± 4.6). Incubation of formulations with L929 cell line proved the biocompatibility of the excipients and non-toxic composition of formulations.

**Conclusion:**

The IgG_1_ and trastuzumab demonstrated similar behavior in spray drying process. The combination of excipients containing trahalose, hydroxypropyl beta cyclodextrin and beta cyclodextrin with proper ratio improved the physical and chemical stability of both IgG_1_ and monoclonal antibody.

## Background

Monoclonal antibodies as an important part of therapeutic proteins are approved for treatment of various chronic and life threatening diseases. Their specific action and relatively low side effects have contributed to vast application of antibodies in many diseases [1]. The majority of antibodies are administrated by injection ordinarily; however, new antibodies and new administration routes such as inhalation [2], transdermal [3], vaginal [4], oral [5] and nasal [6] are investigated broadly as new delivery systems.

The main concern in formulation of antibodies is the intrinsic instability of these labile compounds. Generally, proteins are sensitive molecules to various physical instabilities like denaturation, aggregation, fragmentation and chemical reactions such as deamination, oxidation and isomerization [7,8]. Embedding of protein in dry matrix improves the stability versus physical and chemical degradation by various mechanisms [9-11].

Spray drying as a one-step process has been widely studied in production of dry powders containing proteins [12]. As a limitation, proteins as like as antibodies could be destabilized due to the high temperature and pressures in this process. In this way, the effects of various excipients [9,12] and process variables [12,13] have been investigated in lots of studies [9,10,12,13]. In our previous study, D-optimal design was conducted to optimize IgG_1_ formulation in the presence of sugars and cyclodextrins in order to understand how the spray drying affects the antibody. But as like as many other researches, these efforts have been focused on the processing of IgG as a model antibody and extension of the results of a general antibody to the monoclonal antibodies remains as a challenge. To the extent of our knowledge, no systematic study has been reported as a comparison between these two categories of antibodies. In the present work, trastuzumab as a monoclonal antibody was formulated parallel to the IgG_1_ in the presence of sugar based excipients and the physical stability were compared. Trastuzumab is an important humanized monoclonal antibody that is approved in treatment of breast cancer with over-expressed HER2 receptor [14,15]. So, assessment of the biological activity of trastuzumab is supportive to evaluate the conformational stability of protein during the process.

## Material and methods

### Materials

Hydroxypropyl beta cyclodextrin (HPβCD) was obtained from Acros (Belgium) and Beta cyclodextrin (βCD) was purchased from Sigma (USA). Trehalose dehydrate, potassium phosphate dibasic and disodium sulfate were acquired from Merck (Germany). Trastuzumab (Herceptin®) was purchased from Roche Ltd (Hungary) and the chemicals were from sigma (USA). Human IgG_1_ (with molecular weight of about 150 KD) was supplied by Kedrion (Italy). Prior to each investigation, low molecular weight additive of antibody solution was removed by dialysis with deionized water (cut off: 15 kDa).

### Spray drying of antibody formulations

Spray drying was performed on various aqueous solutions of antibody with different excipients according to Table 1. The design of study was in accordance with the findings of previous studies on IgG_1_. A lab scale Buchi-191 spray dryer (Buchi, Switzerland) was employed to obtain the dry antibody powder. The inlet temperature of 100°C, air flow rate of 700 L/h, liquid feed rate of 1.7 mL/min, and aspiration rate of 100% were selected. The resulted dry powders were collected in dry and well closed glass vials and stored at 4°C.

**Table 1:** **Composition of various formulations of IgG**_**1 **_**and trastuzumab**

**Formulation**	**IgG**_**1**_	**Trastuzumab**	**HPβCD**	**βCD**	**Trehalose**
**F**_**1**_	-	1	-	-	-
**F**_**2**_	-	1	1	-	-
**F**_**3**_	-	1	-	1	-
**F**_**4**_	-	1	-	-	1
**F**_**5**_	-	1	1	1	0.26
**F**_**6**_	1	-	-	-	-
**F**_**7**_	1	-	1	-	-
**F**_**8**_	1	-		1	-
**F**_**9**_	1	-	-		1
**F**_**10**_	1	-	1	1	0.26

### Size exclusion chromatography

Size exclusion chromatography (SEC) was conducted to evaluate the percentage of soluble IgG aggregations as one of the most important physical instabilities. In order to separate the monomer from aggregated antibody, a 300 mm TSK 3000 SWXL column (Tosoh Biosep,Germany) was employed. Approximately, 20 μL of each sample containing 2.5 mg/mL was injected. The mobile phase consisted of 0.1 M disodium hydrogen phosphate and 0.1 M sodium sulfate (pH 6.8) with flow rate of 0.5 mL/min. The antibody concentration was measured by a UV detector (Waters, USA) at 280 nm. All experiments were performed in triplicate.

### Sodium dodecyl sulfate polyacrylamide gel electrophoresis (SDS-PAGE)

In order to determine the antibody fragmentation, non-reducing sodium dodecyl sulphate-poly (acrylamide) gel electrophoresis (SDS-PAGE) was performed. Polyacrylamide gel 10% was prepared and diluted sample with concentration of 100 μg/mL was loaded. The samples were mixed with equal volumes of sample buffer before loading. Protein molecular weight marker of Fermentas® (Germany) was used to estimate the sample molecular weight.

### Fourier transformation infrared spectroscopy (FT-IR)

Nicolet Magna spectrometer (USA) was applied to record the infrared spectra. Briefly, 2 mg of each sample was pressed with 200 mg KBr to make a clear tablet. The Jasco Spectra Manager® software (Japan) was used to deconvolution the spectra and detect the changes in secondary structure of antibody. Second derivative spectrum of amide I region (1600–1700 cm^−1^) is a useful indicator to understand the protein structure. The percent of β sheet, α helix and turn was calculated by mixed Gaussian/Lorentzian function considering β sheet structure absorption in 1640 cm^−1^ and 1695–1690 cm^−1^, α helix in 1660–1650 cm^−1^ and turns from 1690 to1665 cm^−1^.

### Circular dichroism (CD)

Circular dichroism spectroscopy (AVIV, USA) was applied to analysis the secondary structure of monoclonal antibody after reconstitution. Proper amounts of processed powder were dissolved in purified water to make 250 μg/mL solutions. Measurement of buffer and antibody samples were performed in far-UV spectrophotometer at 20°C from 195 to 260 nm with the interval of 1 nm. Buffer spectra were substracted from the relevant protein solutions and converted to mean residual ellipticity. Deconvolution was performed to estimate the secondary structure of trastuzumab spectra. The secondary structure was calculated considering the molecular weight of 148 kDa for trastuzumab and a total number of 1328 amino acids.

### Enzyme-linked Immunosorbent Assay (ELISA)

Direct ELISA was performed in order to determine the in-vitro activity of spray dried trastuzumab. Briefly, a 96 wells plate from Nunc (USA) was coated with 6 μg/mL of recombinant human HER_2_ protein in carbonate/bicarbonate buffer over-night. The plate was blocked with 2% w/v BSA for 1 h at 37°C after three times of washing. About 100 μL of samples were added and incubated for 2 h at 37°C. After washing, 100 μL of the goat anti human antibody conjugated with horse radish peroxidase (HRP) was added and incubated at room temperature for 1 h. The bounded antibody was determined with tetramethyl benzemidine (TMB) as the proxidase substrate for 15 min and the concentration was determined at 650 nm by a STAT FAX 4700 ELISA reader (USA).

### Cell culture studies

The human breast cancer cell line SKBr_3_ and human fibroblast L929 were obtained from cell culture collection of Pasteur institute of Iran. SKBr_3_ was cultured in RPMI medium supplemented with 10% of fetal bovine albumins and the L929 cell line was cultured in Dulbecco’s modified Eagle’s medium with 5% of fetal bovine albumin. Both culture mediums contained 1% of L-glutamine, 100 UI/mL of penicillin G and 100 mg/mL of streptomycin and maintained at 37°C in a humid atmosphere of 5% CO_2_. For the experiment, the cells were harvested after brief incubation with trypsin (0.05%, w/v) and EDTA (0.02%, w/v).

Primarily, the cells were incubated with various concentrations of trastuzumab in the range of 10^1^ to 10^6^ ng/mL and the dose–response of trastuzumab on SKBr3 cells was determined. Further examinations were performed on the samples in concentrations of 10^5^ ng/mL. To evaluate the biological activity of monoclonal antibody in various spray dried samples, the proper amount of each sample were dissolved in 100 mL of medium and added to 96-well plates in triplicate with 2 × 10^2^ cells/well. Cell viability was assayed with mono tetrazolium 3-[4,5-dimethylthiazol-2-yl]-2,5-diphenyl tetrazolium bromide (MTT) colorimetric method. Briefly, the cells were exposed to tetrazolium after 48 h incubation with trastuzumab samples. Living cells were able to metabolized tetrazolium to non-soluble formazan, the formazan salts was dissolved and quantified using spectrophotometer plate reader (BioTek, USA) at 540 nm. The negative control was medium without trastuzumab and the positive control included unprocessed standard trastuzumab with determined concentration.

### Statistical analysis

Data are presented as means ± SD and LSD-test was performed to evaluate the differences between groups. The statistical significancy of results is considered with a P value less than 0.05. The graphs have been drawn by GraphPad® Prism 5and Microsoft® Excel 2007.

## Results and discussion

Similar to other proteins, antibodies can undergo degradation pathways during the spray drying process. Protein degradation can reduce the efficient binding of protein to the receptors and decrease the bioactivity. So it is very impotent to ensure the physical stability as well as biological activity of monoclonal antibodies after spray drying or other processes. In our previous study, optimization and characterization of IgG_1_formulations led to use a combination of trehalose, HPβCD and βCD which resulted in protection of antibody structure up to 99.81 ± 0.7%. In the present study, as shown in Table 1, these excipients were employed in order to evaluate the stability of trastuzumab as a monoclonal antibody in comparison with IgG_1_.

### Physical stability of antibody

**Figure 1:**
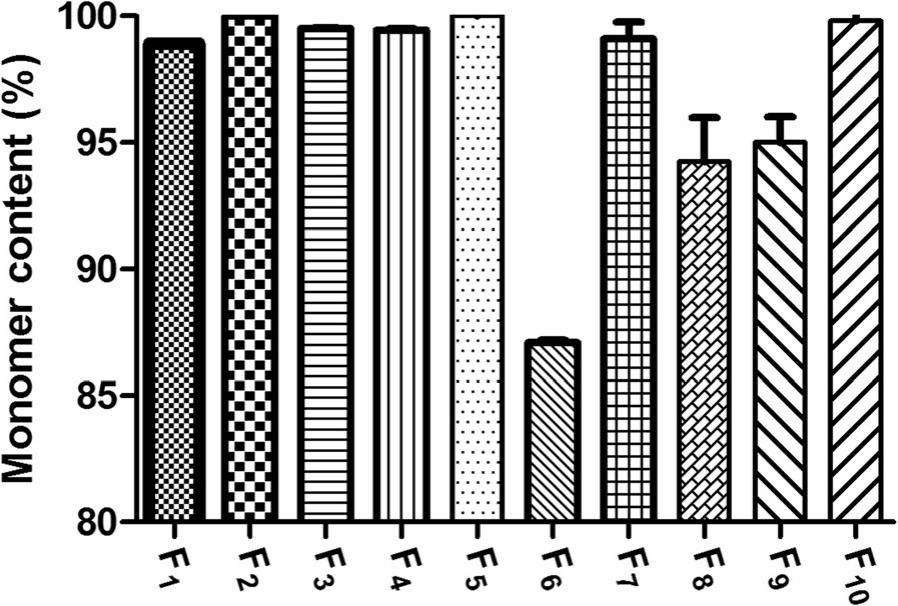
The percent of antibody monomer in microparticles immediately after spray drying analyzed by size exclusion chromatography (SEC).

**Figure 2:**
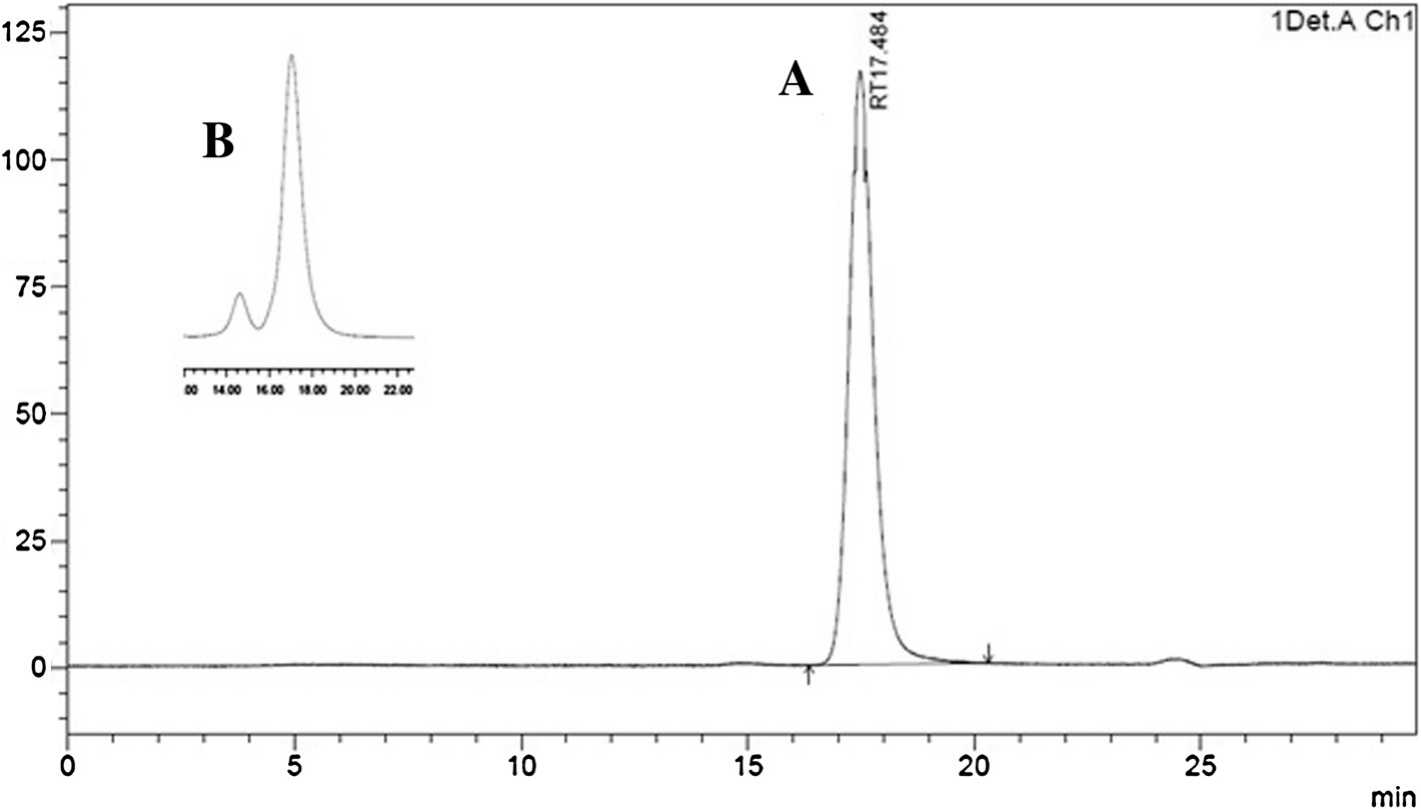
**Size exclusion chromatography of F**_**5 **_**(A); as shown, the aggregation related pick (B) has been disappeared in this formulation.**

The percent of antibody aggregates after spray drying are presented in Figure 1. The pattern of aggregation in IgG_1_ and trastuzumab was similar; however, the percent of aggregations were different. As seen, the pure IgG_1_ contained 87.1% monomer and pure trastuzumab contained 98.9% monomer after process. Application of excipients successfully decreased the aggregation in both of antibodies. In this way, the efficacy of HPβCD was greater than βCD and trehalose. Combination of trehalose, HPβCD and βCD in the F_5_ showed the highest effectiveness in protection of the trastuzumab monomer. As seen in Figure 2, the pick related to the trastuzumab dimmer in SEC chromatogram has been disappeared. This point implicates the synergistic effect of sugars in protection of antibody monomer. Similar observations have demonstrated the synergism effect of various sugars in preservation of proteins by water substitution mechanism [10].

**Figure 3:**
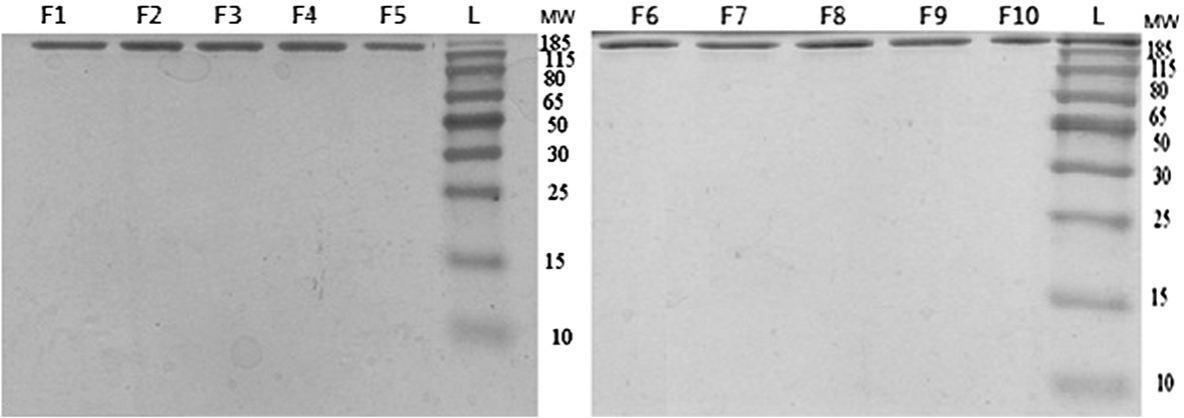
**SDS-PAGE of formulations contained trastuzumab and IgG**_**1**_**.** The standard molecular weights were run in the left of gel.

Non reducing SDS-PAGE was performed in order to reveal fragmentation of antibodies during the spray drying. In this type of SDS-PAGE, reducing agents are not used and consequently, evaluation of protein structure is possible without reduction of di-sulfide bounds. Therefore, detection and quantification of process-related fragmentation would be possible. As shown in Figure 3, all formulations of both trastuzumab and IgG_1_ present bonds just in 150 kDa and there is no distinct bond in lower molecular weight regions. Amphlett G et al. (1996) showed that the bonds such as Asp-Gly and Asp-Pro are more sensitive to cleavage in proteins backbone. So the proteins are prone to cleavage and fragmentation in these places [16]. Moreover, disulfide bonds cleavage lead to formation of antibody fragments of 25 and 50 kDa. If fragmentation took place during the spray drying process, we expected to observe the distinct bonds in SDS-PAGE gel, but the results suggested that process stresses did not affect the trastuzumab backbone. So the data implicated that the short time exposure of antibodies to harsh conditions of spray drying did not induce antibody fragmentation.

**Figure 4:**
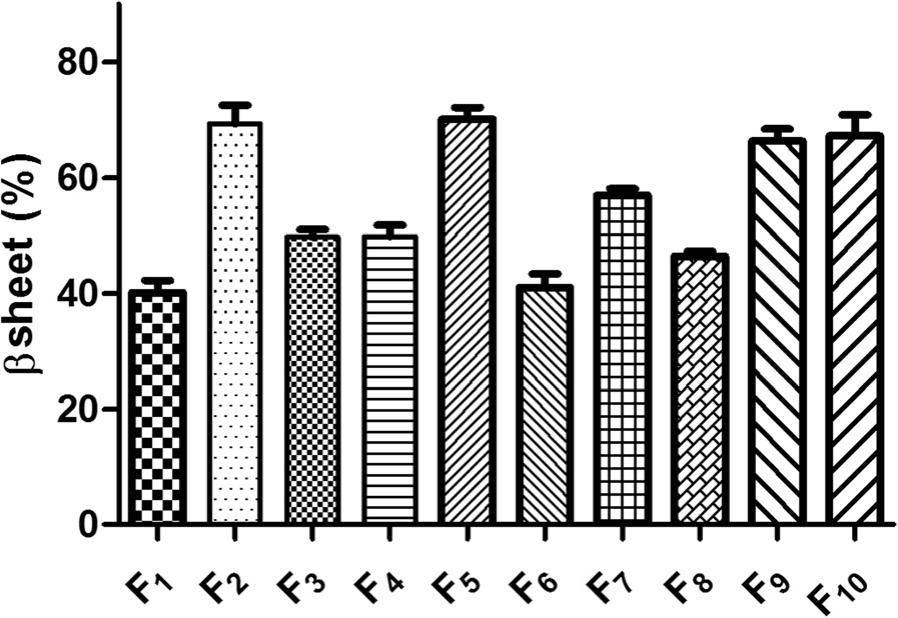
Amount of β structure in dry state analyzed by FTIR in various samples.

The secondary structure of antibody samples was evaluated by FTIR spectroscopy in dry state. As presented in Figure 4, the lowest ratio of β structures (40.13 ± 2.1%) was perceived when pure antibodies were processed by spray drying and presence of excipients in the formulations of antibody increased the β structures content; Where, F_2_ provided 69.35% and F_5_ contained 70.19% of β structure. Processing of pure IgG_1_ lead similarly to 41.01% of β structures and F_10_ as the formulation containing the composition of excipients, preserved the high amounts of β structure after spray drying (67.21%).

**Figure 5:**
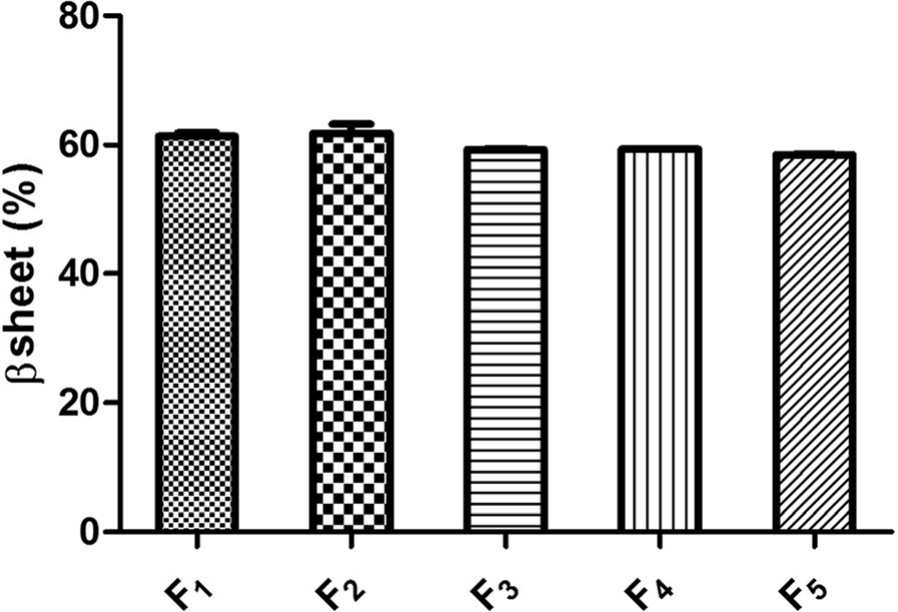
Amount of β structure of spray dried trastuzumab after reconstitution in water analyzed by CD.

The secondary structure of monoclonal antibody, after reconstitution in water, was assessed using far-UV CD spectrophotometer. The amounts of β sheet and β turns in the processed trastuzumab were about 60%. Comparison of FTIR and CD data revealed that dehydration of trastuzumab resulted in substantial and measurable conformational changes and it emphasize that protein structure is highly depended on protein type and formulation conditions, but these changes can be reversible or irreversible. According to the Figure 5, results indicated that trastuzumab reforms to the native structure after reconstitution in water. In agreement, similar studies showed that selection of proper stabilizers minimize the structural changes of proteins such as poly-L-lysine [17], acetate dehydrogenase and phosphor fructokinase [18].

### Biological activity

The biological activity of trastuzumab depends on its ability to target the extracellular domain of human epidermal growth factor receptor protein in tumor cells. Trastuzumab therapy reduces the tumor cells proliferation by several mechanisms. On the one hand, it promotes cell cycle arrest and apoptosis in tumor or metastatic cells and on the other hand, it activates an antibody-dependent cellular cytotoxicity (ADCC) response with the aid of natural killer (NK) cells. The NK cells induce cell death by attaching to the Fc domain of antibody and the activity of trastuzumab highly depends on the ability to contact with HER_2_ antigen as well as NK cells receptors.

**Figure 6:**
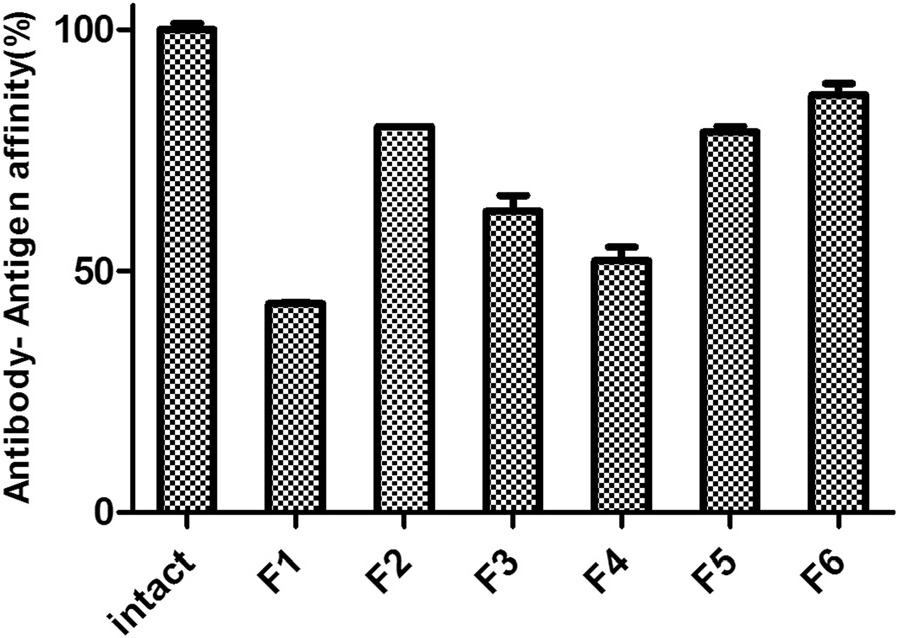
**The Affinity of trastuzumab to Her**_**2 **_**antigen in various spray dried formulations by ELISA study.**

ELISA is an appropriate method for simultaneous evaluation of both F_c_ and F_ab_ fragments of antibody; since, in this analysis method, the structural changes in the antibody are exaggerated. Any change in the F_ab_ or F_c_ can affect the affinity of antibody to the antigen or secondary antibody. Direct ELISA was applied to evaluate the stability of trastuzumab during the spray drying in comparison with unprocessed antibody as positive controls. As presented in Figure 6, just 43.2 ± 0.2% of the pure antibody was quantified after processing; whereas, presence of excipients in F_2_ and F_5_ enhanced the affinity of antibody to the antigen up to 79.9 ± 0.1% and 86.5 ± 2.3%, respectively.

**Figure 7:**
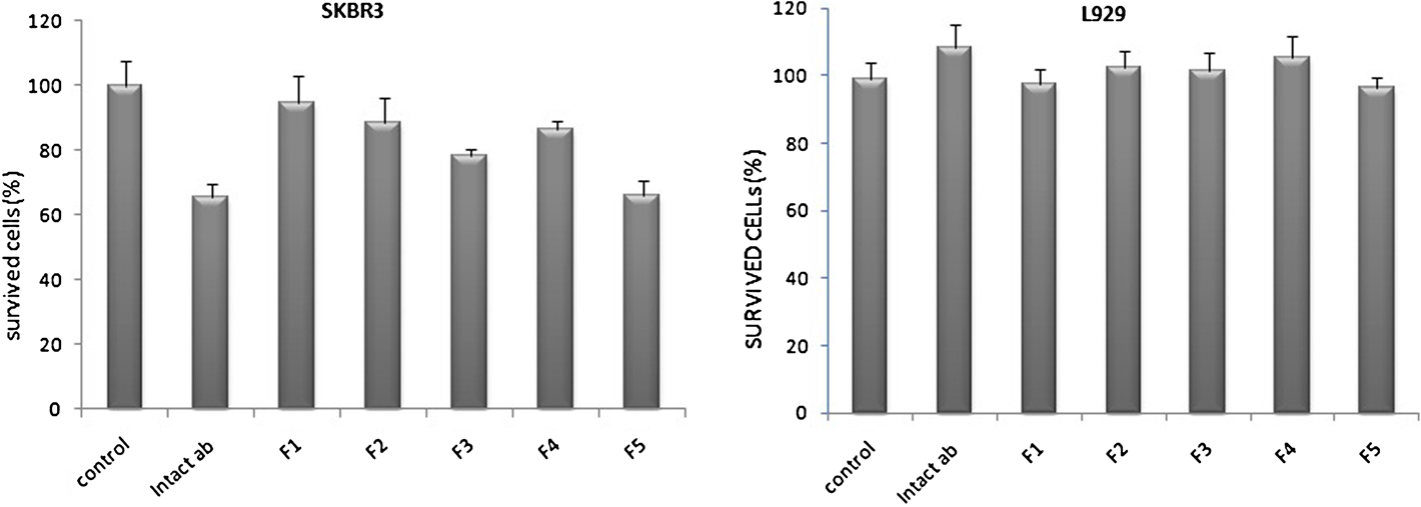
**The survived SKBr**_**3 **_**and L929 cell line after incubation with 10**^**5**^ **ng/mL of trastuzumab in various formulations.**

Furthermore, bioactivity of monoclonal antibody was evaluated by means of HER_2_-over-expressing breast cancer cell line (SKBr_3_). The cells were incubated in the presence of 10^5^ ng/mL of spray dried samples and unprocessed standard trastuzumab as positive control. The cells viability was evaluated by MTT assay after 48 hours. Statistical comparison of survived cells after incubation showed that the ability of F_1_ containing processed pure trastuzumab to kill the cells was the minimum (Figure 7). The 2-side dunnett test indicated no difference between F_1_ and negative control. However, the unprocessed trastuzumab presented a great activity to kill the SKBr_3_ cells (with the minimum cell viability of 65.88 ± 3.95% after incubation). The ANOVA with the aid of post doc (LSD) test among spray dried formulations indicated the equal ability of F_2_, F_3_, F_4_ with F_5_, which showed the greatest ability to preserve the antibody activity (65.99 ± 4.6%). Also, LSD test indicated no difference between F_5_ and positive control. As a result, it could be deduced that combination of various excipients with different properties led to inhibition of aggregation and preservation of antibody structure as well as protection of amino acids in the protein backbone.

Some previous studies indicated the citotoxicity of cyclodextrine and other derivatives on the body cells or on red blood cells [19,20]. The L929 is a fibroblast cell line L929 without any HER_2_ antigen on the surface that is suitable for evaluation of toxicity and biocompatibility of formulations. The same concentration of trastuzumab was incubated with this cell line and analysis of cell viability in the presence of different formulations showed no toxic effect at concentrations of 10^5^ ng/mL and the 100% of cells were alive after incubation with various formulations after 48 hr (Figure 7). Consequently, we concluded that the death in SKBr_3_ cells were due to the active trastuzumab rather than formulation excipients. Also the biocompatibility of formulations was confirmed in this experiment.

## Conclusion

This report focused on physicochemical stability and biological activity of antibodies after spray-drying. Combination of HPβCD, βCD and trehalose provided the maximum efficacy in protection of the IgG_1_ as a general antibody and trastuzumab as a monoclonal antibody. Coexistence of these excipients in the formulations preserved monomers up to 99.1% and 99.9% for IgG and trastuzumab, respectively.

The conformational changes of processed antibodies in dry structure were reversible and it could reform to the native stricter after reconstitution in water. The activity of trastuzumab in best formulation was detected up to 86.46% in ELISA test and it was confirmed by cell culture studies.

## Competing interests

The authors declare that they have no competing interests.

## Authors’ contributions

VR performed the experiment and prepared the manuscript; AV and ARN supervised the project and helped in study design; MASH gave consultation on cell culture and analyzing the antibody bioactivity; AKH gave consultation on ELISA assay, MS helped in statistical analysis and preparation of the manuscript. All authors read and approved the final manuscript.
